# Supplementary figures and images for: Differential Recovery Patterns of the Maxilla and Mandible after Eliminating Nasal Obstruction in Growing Rats
Source: J Clin Med. 2022 Dec 11;11(24):7359. doi: 10.3390/jcm11247359 (PMC9783669; doi:10.3390/jcm11247359)

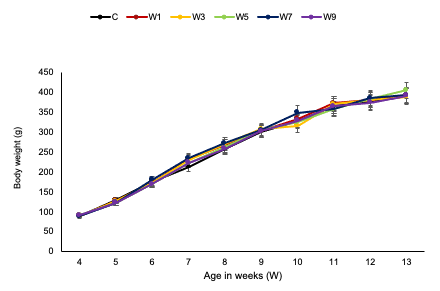

Supplement: Supplementary file 1 [file jcm-11-07359-s001.zip › Figure S1.tiff]

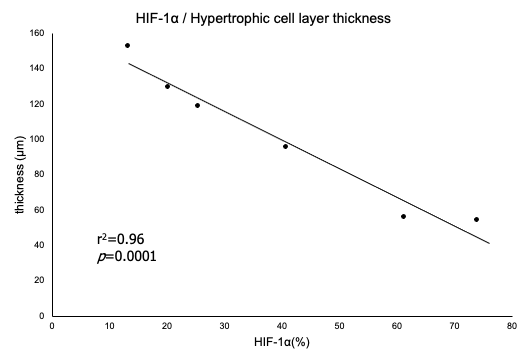

Supplement: Supplementary file 1 [file jcm-11-07359-s001.zip › Figure S2.tiff]

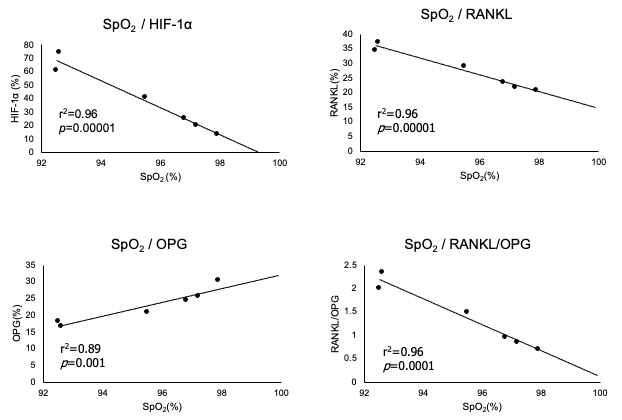

Supplement: Supplementary file 1 [file jcm-11-07359-s001.zip › Figure S3.tiff]
